# Artificial intelligence enabled applications in kidney disease

**DOI:** 10.1111/sdi.12915

**Published:** 2020-09-13

**Authors:** Sheetal Chaudhuri, Andrew Long, Hanjie Zhang, Caitlin Monaghan, John W. Larkin, Peter Kotanko, Shashi Kalaskar, Jeroen P. Kooman, Frank M. van der Sande, Franklin W. Maddux, Len A. Usvyat

**Affiliations:** ^1^ Maastricht University Medical Center Maastricht The Netherlands; ^2^ Fresenius Medical Care Waltham MA USA; ^3^ Renal Research Institute New York NY USA; ^4^ Icahn School of Medicine at Mount Sinai New York NY USA

## Abstract

Artificial intelligence (AI) is considered as the next natural progression of traditional statistical techniques. Advances in analytical methods and infrastructure enable AI to be applied in health care. While AI applications are relatively common in fields like ophthalmology and cardiology, its use is scarcely reported in nephrology. We present the current status of AI in research toward kidney disease and discuss future pathways for AI. The clinical applications of AI in progression to end‐stage kidney disease and dialysis can be broadly subdivided into three main topics: (a) predicting events in the future such as mortality and hospitalization; (b) providing treatment and decision aids such as automating drug prescription; and (c) identifying patterns such as phenotypical clusters and arteriovenous fistula aneurysm. At present, the use of prediction models in treating patients with kidney disease is still in its infancy and further evidence is needed to identify its relative value. Policies and regulations need to be addressed before implementing AI solutions at the point of care in clinics. AI is not anticipated to replace the nephrologists’ medical decision‐making, but instead assist them in providing optimal personalized care for their patients.

## 1 INTRODUCTION

Artificial intelligence (AI) is anticipated to transform health care through advancements in clinical decision support. Rapid advancements in computational power and improvements in statistical techniques ultimately enable AI to be leveraged to identify hidden interactions and patterns within large, complex, multi‐level datasets. AI has been suggested as the next natural progression of traditional statistical techniques (eg, logistic regression, linear regression, etc), and these analytical advancements can be applied to the practice of medicine.^1^, ^2^ An AI‐based “virtual coach” using a diverse set of inputs and algorithms may have the potential to aid in personalized medical guidance for patients.^3^ AI medical decision support tools for clinicians may also improve efficiency by optimizing routine workflows and aid them in the process of providing care.^4^

In a recent bibliometric study on the global evolution of AI in health care and medicine, it is shown that clinical applications of AI are relatively common in fields like ophthalmology, oncology, and cardiology.^5^ However, the use of AI is scarcely reported in nephrology, despite attributes of large datasets^6^ and one of the highest disease burdens.^7^ In‐center hemodialysis (HD) is typically performed three times per week for 3‐5 hours, thus amassing a large volume of clinical data captured in electronic medical records (EMR). These large treatment datasets are ideal for AI applications. With advances in technology, remote treatment monitoring applications allow clinical data to be collected from patients dialyzing at home. Recently, it has also become possible to measure and store beat‐to‐beat hemodynamic and respiratory values during dialysis treatment.^8^ Furthermore, the emerging field of medical grade wearables is anticipated to yield even more robust data in all populations.^9^

The aim of this review is to: (a) provide an overview of the AI application process in a clinical setting; (b) provide brief descriptions of select advanced Machine Learning (ML) algorithms; (c) present the current status of AI in research toward kidney disease and dialysis; and (d) explore future pathways for AI within the discipline of nephrology. This review focusses on the applications of AI in progression to end‐stage kidney disease (ESKD) and dialysis omitting the unique acute kidney injury population.

## 2 TYPES OF AI

**FIGURE 1:**
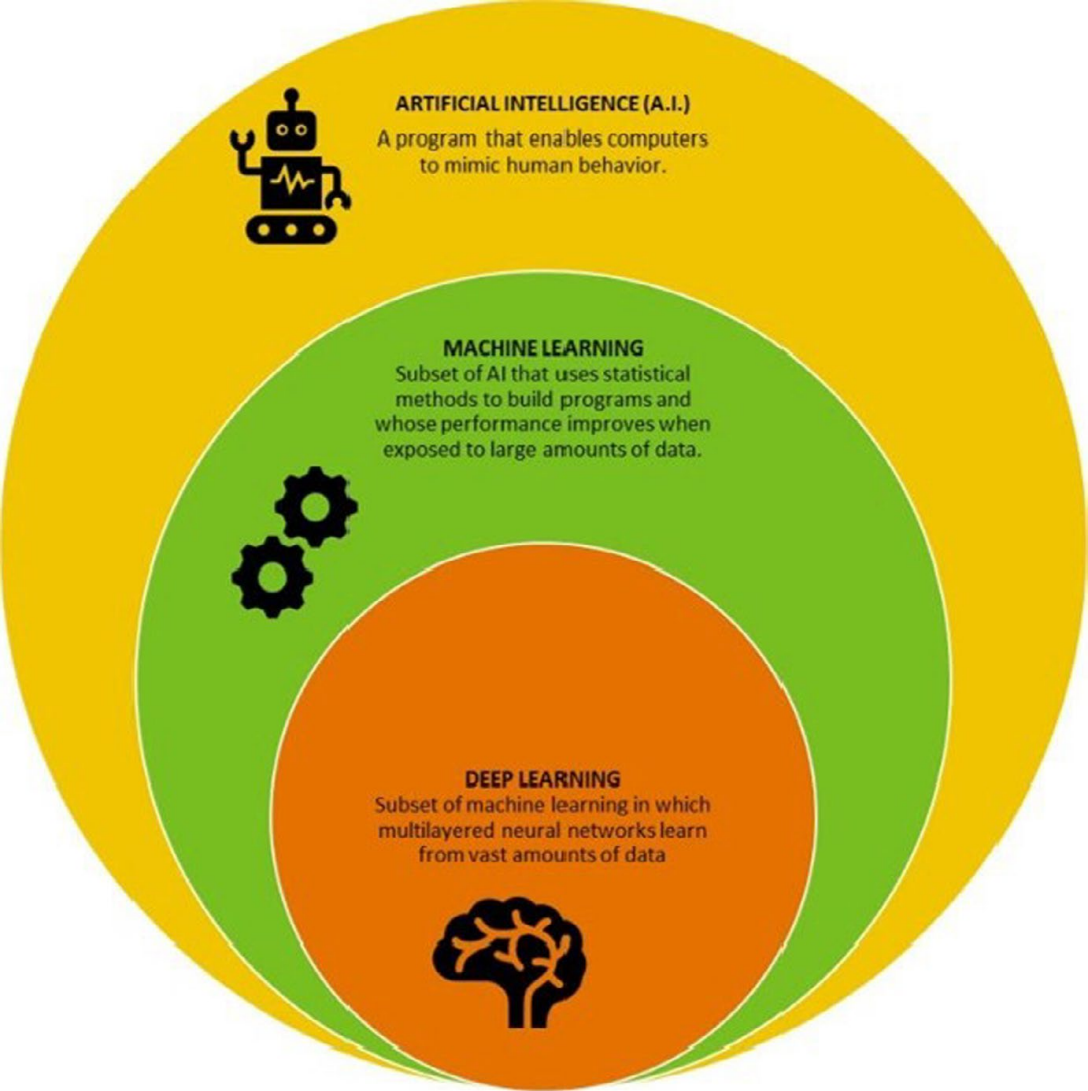
Figure shows the relationship between artificial intelligence (AI), machine learning (ML), and deep learning (DL). ML is a subset of AI and DL is a subset of ML. ML is a sub‐discipline of AI that uses training examples of how to perform a specific task without explicit instructions to identify associations for a given outcome measure. DL is a subfield of ML that mimics neural networks to learn

There is no universal definition of AI, but central to most definitions is the ability of a learning system to mimic human behavior. As depicted in Figure 1, AI is an umbrella term that brings together concepts from several fields such as computer science, statistics, algorithmic, ML, information retrieval, and data science at large.^10^ ML techniques are very powerful in their ability to detect hidden patterns in large datasets that are otherwise difficult to identify by traditional statistical techniques.

**FIGURE 2:**
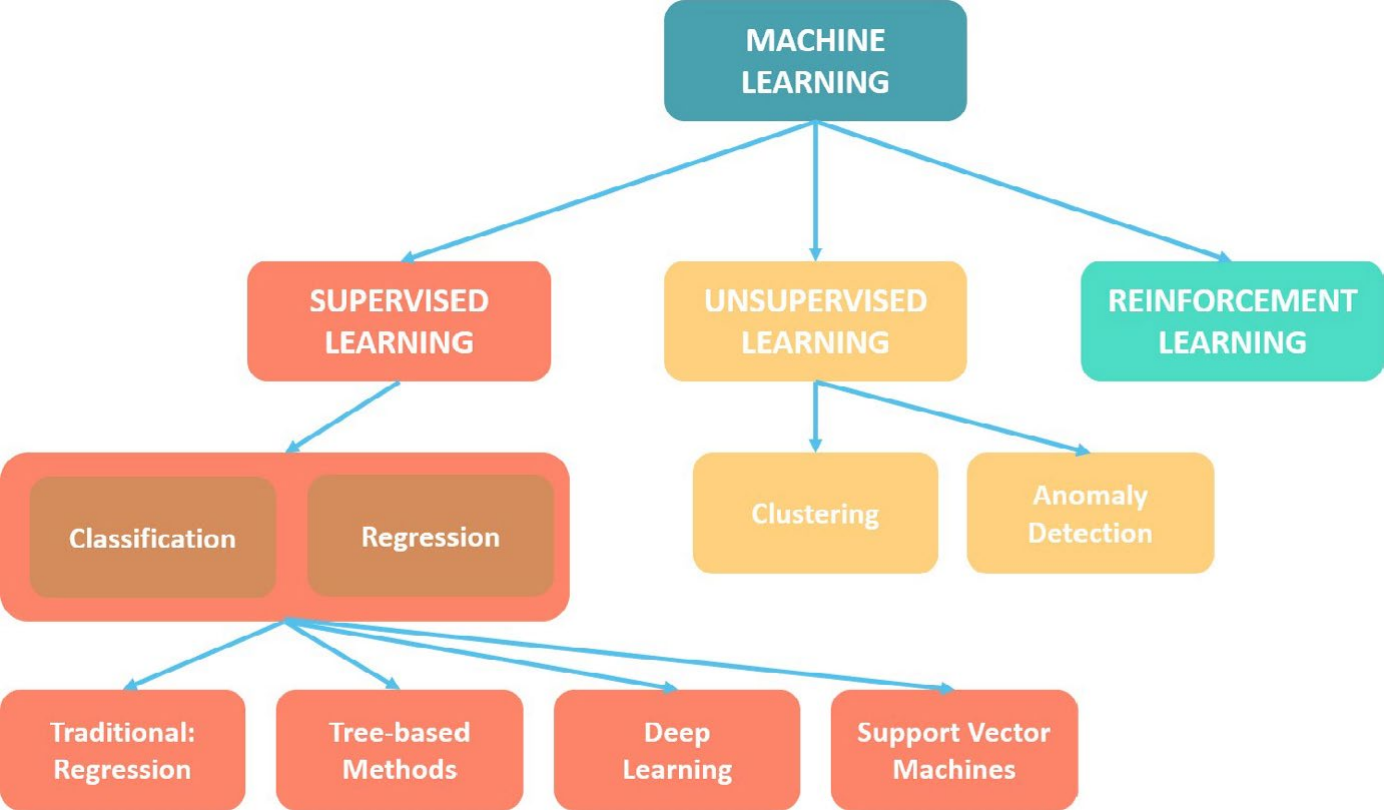
Supervised learning (SL) and unsupervised learning (UL) are the two main categories of machine learning (ML). Deep learning (DL) is a subset of ML. SL algorithms are used to learn the optimal parameters of the predictive model by investigating past examples with known inputs and known outputs. UL algorithms learn about patterns in the input data itself and does not have a known output

The types of ML techniques that currently exist for building AI applications broadly fall into three families (Figure 2), namely supervised learning (SL), unsupervised Learning (UL), and reinforcement learning (RL). SL and UL are briefly discussed below, although technical details are beyond the scope of this review.^11^ Most of the applications of RL are in the fields of board and video games and beyond the scope of this paper.

### 2.1 Supervised learning

Supervised learning is the most frequently used type of ML. The objective of SL is to build a predictive model that takes historical input features to predict a specific output. For example, one may want to predict if a patient will miss their next dialysis treatment (binary output Yes/No) or predict how long it would take until a patient will transition to dialysis (continuous output).

Supervised learning can be divided into two categories (classification and regression) depending on the type of the output (Figure 2). In classification, the output belongs to a set of distinct classes (eg, missed treatment vs not missed treatment). In regression, the output is usually a continuous numerical quantity (eg, N days until transitioning to dialysis).

There are many ML algorithms for building predictive models ranging from traditional to more advanced methods. Prediction performance of these models is usually presented as area under the receiver operating characteristic curve (AUROC).^12^ The most common traditional SL methods are logistic regression (for classification) and ordinary least squares regression.^13^ These traditional methods are popular analysis techniques within health care and hence not discussed here for brevity. Over the past decade, more advanced techniques, such as tree‐based methods and deep learning (DL) algorithms, have grown in popularity.

The foundation of tree‐based methods is the decision tree, a ML technique for sequentially dividing the samples based on determining if a selected feature is greater than, or less than, a threshold determined by the model. At every level of the decision tree, the ML model learns which feature to use, and which threshold is the best. Unfortunately, a single decision tree can memorize the training data, resulting in poor performance on unseen data. As a result, many advanced analytical techniques (eg, random forest and Gradient Boosting Classifier) have been created to improve upon traditional single decision trees, increasing generalization to new data.^14^ In random forest methods, multiple decision trees are created using random subsets of samples (ie, by bootstrapping) and random subsets of the input features (ie, bagging). On the other hand, Gradient Boosting methods sequentially add decision trees with few levels of nodes (shallow) that leads to a progressive improvement in model performance. One Gradient Boosting method known as XGBoost is currently one of the top performing models in the ML field.^15^

An extensive bibliography of new SL techniques, their application, and performance compared to traditional techniques is becoming available. Akbilgic et al compared several different ML modeling techniques to predict risk of death in incident dialysis patients.^16^ The random forests model outperformed logistic regression with an AUROC of 0.76 compared to an AUROC of 0.68.

**FIGURE 3:**
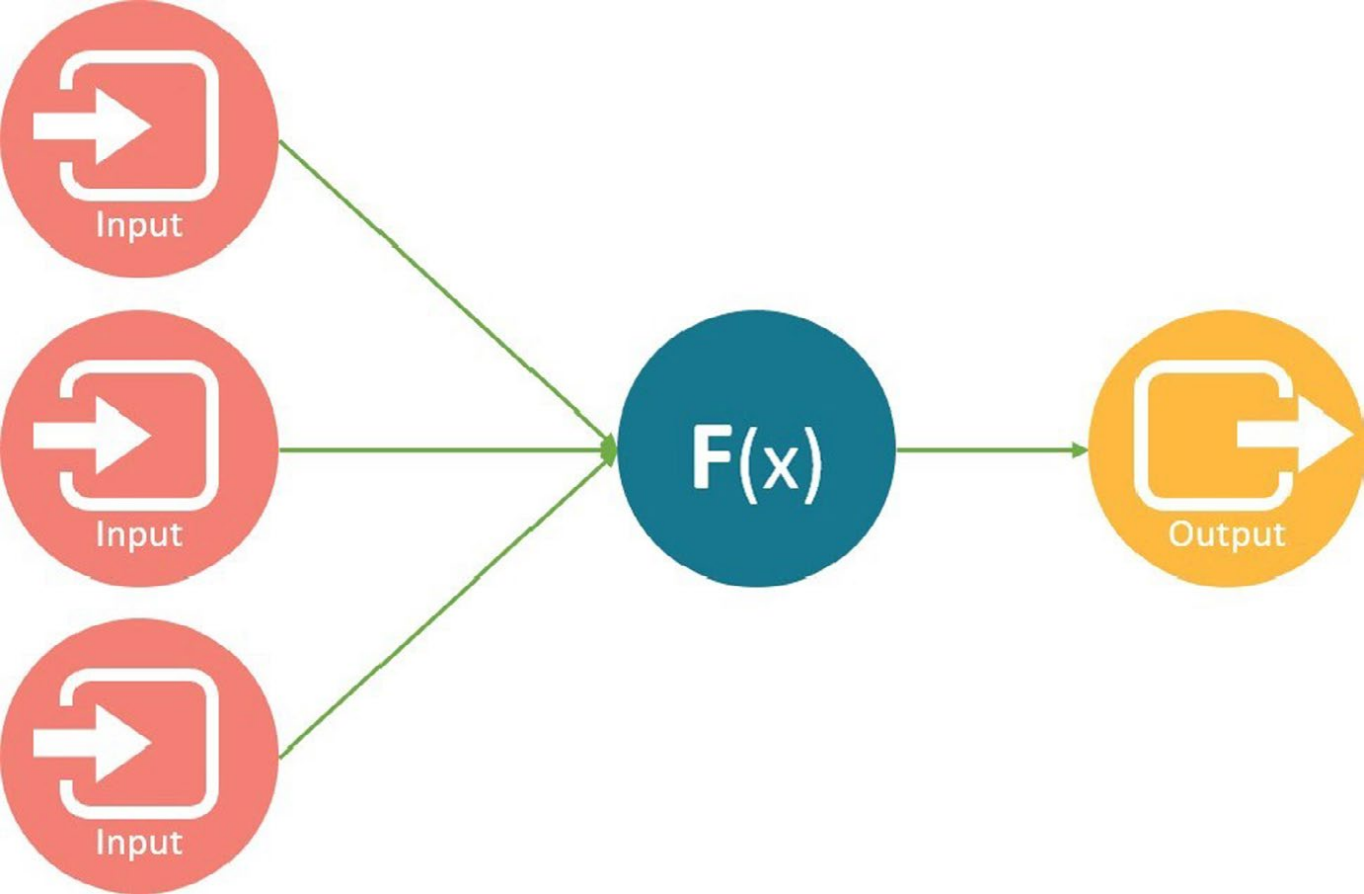
A very simple artificial neural network (ANN) with an input layer comprised of three inputs, hidden layer comprised of one neuron, and the output layer. ANN's neuron usually combines input from multiple sources through nonlinear activation functions

**FIGURE 4:**
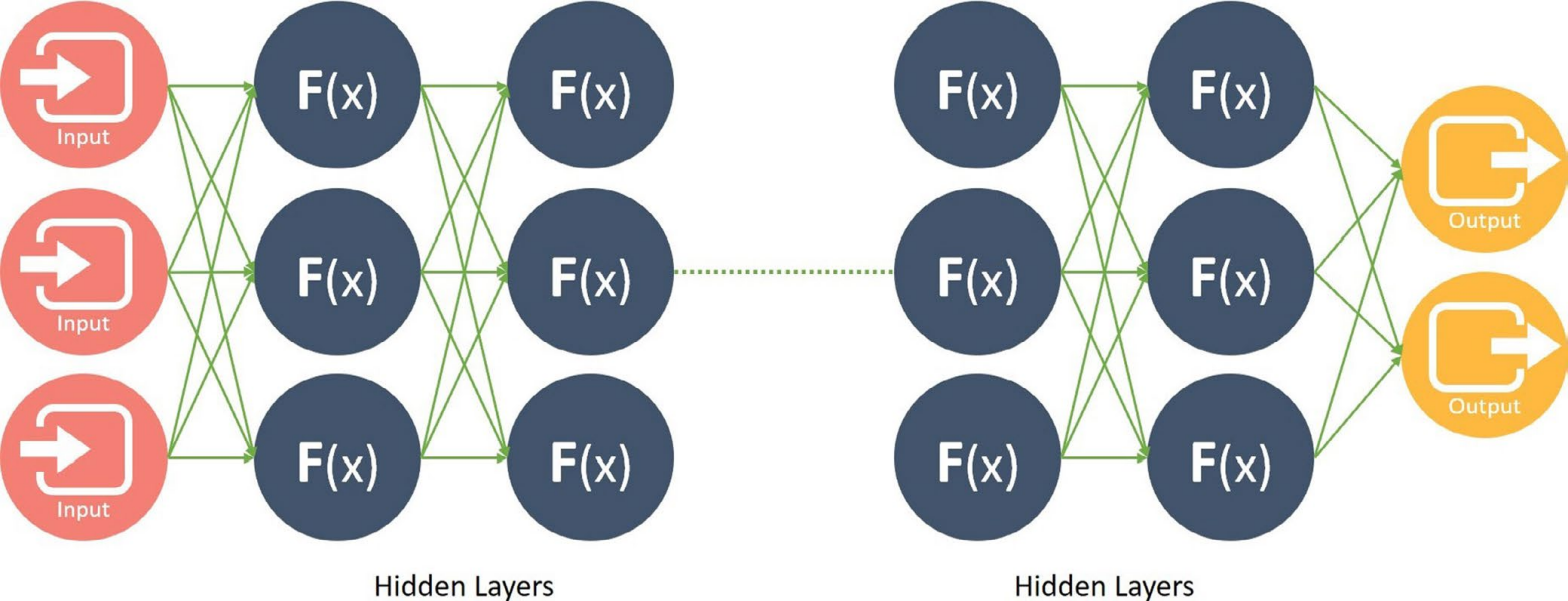
Deep Learning Network. Input layer with three inputs, multiple hidden layers of neurons, and two output layers. Higher the number of hidden layers deeper is the network

Deep learning, which uses artificial neural networks (ANNs), is another SL technique that has grown in popularity in the last decade. ANN began in the 1950s with the MADALINE algorithm,^17^ but it was not until recently with advances in computational power that ANN/DL could be computed in a reasonable time. The name ANN refers to its core functional unit, neuron (Figure 3). ANN's neurons usually receive multiple inputs that are mathematically combined through nonlinear (eg, sigmoidal) activation functions. A simplest neural network is the standard logistic regression. On the other hand, DL consists of stacking multiple layers of these units in the hidden layer (Figure 4). These layers connect to units of an output layer serving as the final output of the model.

The weights of the inputs are the parameters learned in ANN throughout the entire neural network. Given a set of weights, the training input features are fed forward through the neural network to create a set of predictions. The predictions are then compared to the actual output labels and this difference (ie, the error) is fed backward through the hidden layers. Over several iterations, the network “learns from its mistakes” and optimally adjusts its unit weights to a point where it can accurately predict the outcome.

**FIGURE 5:**
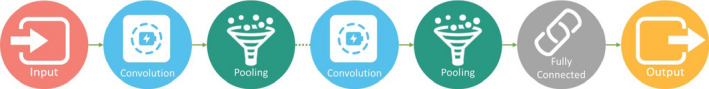
Convolution Neural Network (CNN) is a class of deep learning neural networks that is widely used for image classification. A CNN includes an input layer (image data), multiple hidden layers (convolution to extract features, pooling for subsampling features, and fully connected layer to classify images), and an output layer

**FIGURE 6:**
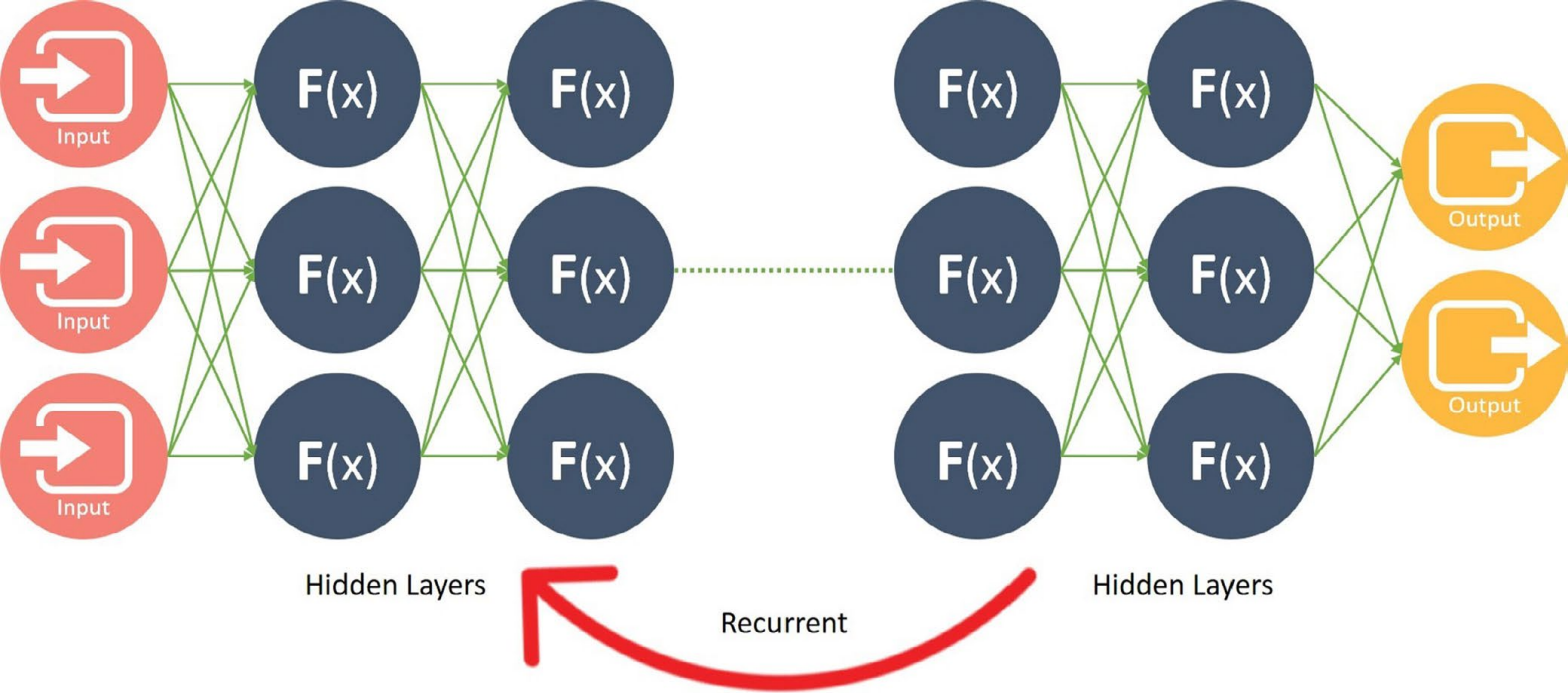
Recurrent neural network (RNN). In RNN, the output from the function is fed back in the model in order to minimize error

To optimize these weights, the DL algorithm uses a technique known as backpropagation which was invented in the 1980s.^18^ As the number of layers are added to the neural network, the number of weights and connections increase dramatically. Convolutional neural networks (CNNs) and recurrent neural networks (RNNs), as shown in Figures 5 and 6, are two variants of ANN that have also been created to reduce the number of weights, resulting in increases in performance, and decreases training time. A CNN is mostly used for image processing and RNN is widely for natural language processing (NLP).^19^

In the medical field, DL^20^ (specifically CNN) has been mainly applied for image processing in the fields of radiology, histology, dermatology, and retinopathy, which has been able to demonstrate at or above clinical performance.^21^, ^22^, ^23^ For example, in cardiology, DL has been used to predict outcomes after cardiac arrest.^24^

Support vector machine (SVM) is a form of SL, where the ML algorithm performs complex data transformations on the labeled data and defined output to draw boundaries within the input data. SVMs can be used to solve classification problem as well as a regression problem.^25^

### 2.2 Unsupervised learning

In UL, there is no output label, but rather the objective is to learn about patterns in the input data itself. UL techniques usually focus on clustering, dimensionality reduction, or anomaly detection.^26^ A commonly used clustering technique is k‐means clustering.^27^ k‐means clustering utilizes an iterative refinement algorithm with assignment step and update step to partition the data into k clusters, and the algorithm aims to minimize the within‐cluster variance and maximize the between‐cluster variance. It is critical to determine an appropriate number of k clusters when using k‐means clustering method. Hierarchical clustering^28^ is another commonly used clustering technique that usually creates a hierarchy of clusters from top to bottom. For example, using hierarchical clustering, Liu et al identified clusters of US states based on unhealthy behaviors, preventive measures, and CKD‐related outcomes in adults living in cities.^29^ They concluded that such information may be of interest to policy makers to understand sociodemographic factors and other risk factors could contribute to the prevalence of CKD.

Table 1 shows a very high level overview of the differences between the traditional statistical techniques and advanced analytical methods.

**TABLE 1:** Differences between traditional statistical methods vs advanced analytical techniques

Factors	Traditional statistical techniques	Advanced analytical techniques
Training data	Works with Smaller Datasets	Better with Large Datasets
Usability	Exploratory and baseline analysis	Iterative, complex, and ready to be deployed in clinical application
Interpretability	Easily interpretable	Complex techniques can be difficult to interpret
Hardware and training time	Requires simple hardware configuration and less training time	Complex models require powerful computing hardware and more training time
Types of input data	Works well only with categorical and numerical data	Works with all types of data including audio, image, free text
Examples	Logistic and Linear Regression, Generalized Additive Models, single decision tree	Neural Network, complex decision trees with several layers

## 3 AI APPLICATION PROCESS

**FIGURE 7:**
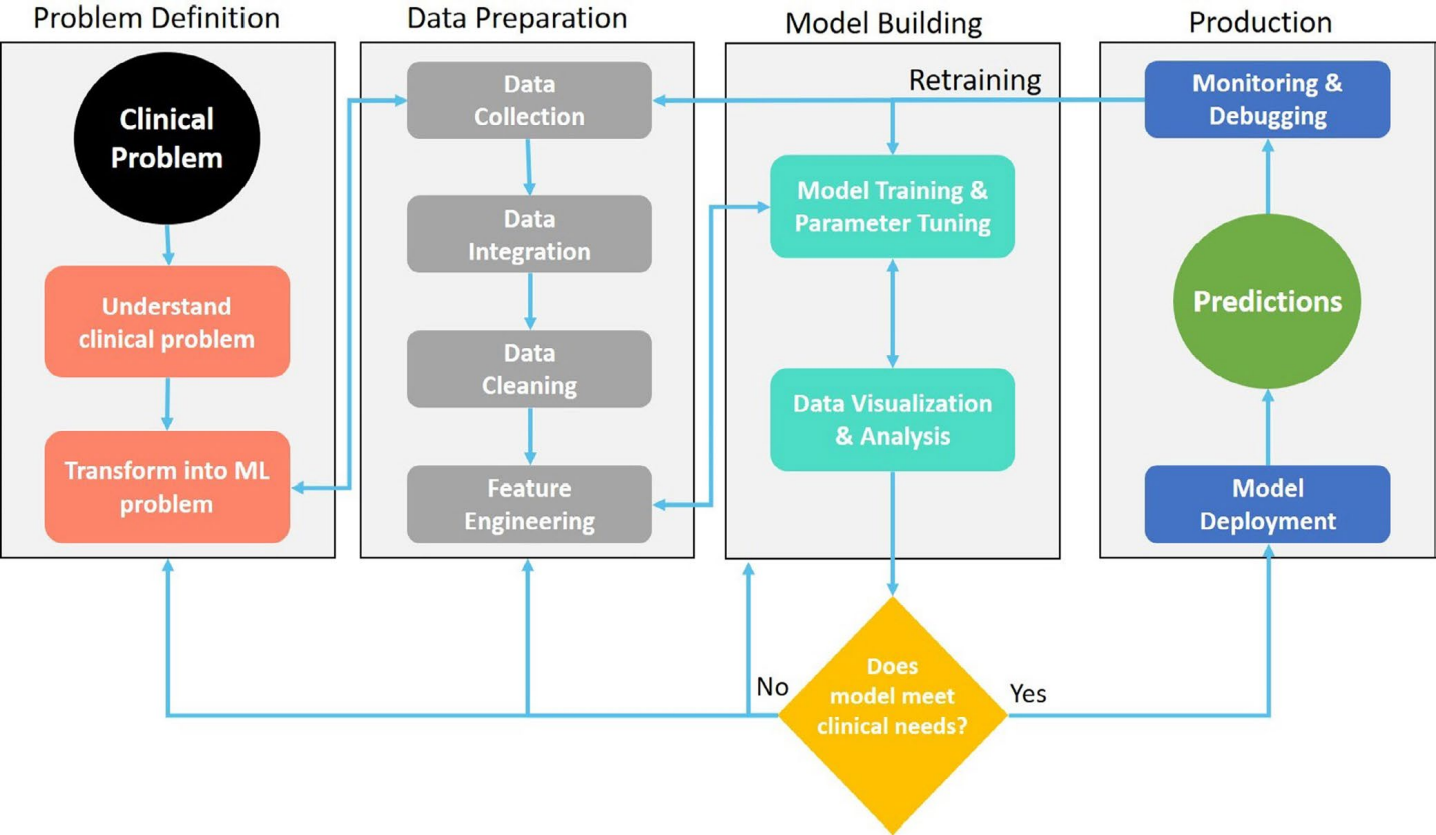
Process for application of artificial intelligence with four phases: Problem definition, data preparation, model building, and production

The AI application process in a clinical setting generally consists of a series of stages (Figure 7). For ML, the process begins by defining the problem. This includes understanding the context of the clinical problem at hand and transforming the clinical problem into a relevant ML problem.

The next stage consists of understanding the quality and quantity of the clinical data available and preparation for modeling. Data preparation consists of collection, integration, cleaning, and using clinical knowledge to build predictors (feature engineering) for the ML model. In HD, the enormous amount of EMR data collected at the point of care provide a rich platform to employ ML. ML thrives on processing a huge number of variables combing them in nonlinear interactive ways. This capability allows new kinds of data (eg, free text, images, videos, sound, and temporal data) to be utilized. The volume and complexity of such data add additional challenges in analyzing the data.

With a set of well‐engineered features, the predictive models are trained and tuned until acceptable performance is achieved. It is anticipated that some steps of the process have bi‐directional arrows because they can result in modifications for previous steps (Figure 7). If the model meets the needs of the clinical problem, the trained model can be deployed in production. During production, it is advisable to monitor the predictions and retrain the model when necessary.

## 4 APPLICATIONS IN KIDNEY DISEASE

There are several unmet needs in nephrology and there is a huge potential for use of big data and AI in patients with kidney disease. The applications of AI kidney disease can be broadly subdivided into three main topics: (a) predicting events in the future; (b) treatment and decision aids; and (c) identification of existing, but unrecognized, patterns. Table 2 shows a summary of key AI‐related studies that have been published in the field of kidney disease. Currently, only one published study reports the clinical application, although the use of AI in kidney disease is reported more commonly in conference abstracts suggesting that the scientific community has more contributions on the horizon.

**TABLE 2:** Key publications of AI applications in kidney disease

Author, year	AI techniques	No of patients	Outcome predicted	Performance	Clinical application use
Akbilgic et al^16^ 2019	Random Forest	27 615	Risk of death	AUROC: 0.70‐0.76	NA
Goldstein et al^32^ 2014	Random Forest	826	Sudden cardiac death	AUROC: 0.78‐0.79	NA
Mezzatesta et al^33^ 2019	Support Vector Machine	1216	Cardiovascular disease	Accuracy: 92.15%‐92.25% AUROC: 0.50‐0.74 Precision: 72%‐89% Recall: 73%‐94%	NA
Chauhan et al^39^ 2020	Random Forest	1369	CKD progression	AUROC: 0.77‐0.80 PPV: 62% in high‐risk group NPV: 92%‐96% in low‐risk group	NA
Norouzi et al ^42^2016	Artificial Neural Networks	465	CKD progression	MSE: 58.63‐64.00 MAE: 4.77‐5.93 NMSE: 4.77%‐4.88%	NA
Barbieri et al ^46^2016	Artificial Neural Networks	752	Anemia management	MAE: 0.59 g/dL	Yes
Zhang et al^74^ 2017	Random Forest	83	Immune fingerprints	AUROC: 0.993 Sensitivity: 98.5% Specificity: 92.6%	NA

Abbreviations: AI, artificial intelligence; AUROC, area under the receiver operating curve; MAE, mean absolute error; MSE, mean square error; NMSE, normalized MSE; NPV, negative predicted value; PPV, positive predicted value.

### 4.1 Predicting the future

#### 4.1.1 Predicting outcomes

Patients with ESKD have high mortality as well as hospitalization rates.^7^ Prediction models can assist with early care planning and triaging resources where there is potential for the greatest clinical benefit. Interventions performed based on predictions would be specific to the outcome and may warrant ad hoc and/or extra evaluations and clinical screenings in addition to routine care. Early mortality and hospitalization prediction models using traditional statistical techniques built on a select number of features have been reported.^30^ The AUROC for traditional statistical mortality and hospitalization prediction models usually fall in the range between 0.65 and 0.75.^31^ In nephrology, prediction of sudden cardiac death in older HD patients was an early example of employing an advanced ML method where a random forest model yielded a AUROC of 0.79.^32^ In another example, Mezzatesta et al used SVM, to predict the risk of ischemic heart disease in dialysis patients with an accuracy of approximately 92%.^33^

Recent studies show a large set of features and their interactions with other features can be employed using advanced ML methods to better estimate potential risk factors preceding mortality and/or hospitalization.^34^ For instance, a large dialysis organization (LDO) of Fresenius Medical Care (FMC), an integrated kidney disease care organization, has developed and deployed a predictive model that includes more than 200 variables to identify patients treated with in‐center HD who have an increased risk of hospitalization in the next 12 months. The model is built using XGBoost classifier with an AUROC of 0.81.

As part of a pilot study reported in a congress abstract, the LDO has suggested use of the predictive model to assist clinicians with targeting additional interdisciplinary assessments and interventions resulted in a decrease of the average yearly hospital admission rate and average yearly hospital days rate compared to controls in the neighboring region that did not participate in the pilot and receive predictive model reports.^35^ Such prediction model appears to have the potential to provide an intelligent method of triaging additional resources in dialysis clinics.

Advanced AI applications are powerful in analyzing vast amounts of clinical data to look for subtle changes in a patient's condition or worsening status for short‐term outcomes. Dial disorders/disease exacerbations sometimes exhibit clear symptoms in the days prior to an event; however, the occurrence of minor signals of a worsening condition that do not clearly warrant any immediate intervention or appear unrelated to the cause of the event that takes place soon after can be a clinical challenge. Recent efforts by the LDO reported in a congress abstract led to the development and implementation of a model to predict imminent hospitalizations in ESKD patients who are at risk of getting hospitalized within the next 7 days.^36^ The model uses over 1500 variables from a range of data sources (eg, treatment vitals, laboratory measurements, comprehensive assessments, and nursing clinical notes). The unstructured clinical notes are converted into numerical data using NLP techniques, specifically word2vec and CNN. The output of the CNN is then combined with other structured numerical data to train an XGBoost tree classifier. The final model has an AUROC of 0.78. As reported in another congress abstract, this model is currently used by a team of nurses and has improved their workflow significantly.^37^ Although the effectiveness of this imminent hospitalization model and subsequent interventions is unknown and being evaluated, its potential to assist clinicians with near real‐time insights of risk levels and predictors driving the risk determination is promising and could help them with targeting interventions and transitional care planning before and after hospitalization episodes.

#### 4.1.2 Predicting chronic kidney disease progression

Chronic kidney disease (CKD) is a growing health crisis across the world.^7^ Detecting it early and managing the progression of the disease are critical for positive patient outcomes and controlling health‐care costs. Due to challenges in understanding the trajectory of this disease, providing care planning before initiation of dialysis and helping patients make appropriate vascular access and modality choices may be difficult.

Traditional and AI techniques are being developed to predict CKD progression. Tangri et al have developed a traditional regression model for prediction of kidney failure from CKD stages using demographic, clinical, and the most recent clinical data from two independent cohorts of CKD patients stages 3‐5.^38^ In two other recent studies, random forest models have been developed to generate a prognostic risk score by combining data from EMR and circulating biomarkers, such as plasma tumor necrosis factors and kidney injury molecule‐1, to predict CKD progression.^39^, ^40^ The AUROC in one of the studies by Chauhan et al was 0.77‐0.80. Xiao et al compared several ML methods to predict the risk of proteinuria >1 g/d in CKD patients using demographic data and blood biochemical features.^41^ In this case, the traditional logistic regression model outperformed other ML models with AUROC 0.87. They conclude that advanced ML models are best when the amount of data is large, whereas linear models perform better in relatively smaller datasets. On the other hand, Jamshid Norouzzi et al developed an ANN to predict renal failure progression in patients with CKD. The model could accurately (>95%) predict the estimated glomerular filtration rate (eGFR) in 6, 12, and 18 months interval.^42^

As reported in a congress abstract, the Renal Research Institute used data from 28,608 patients with CKD from 2000 to 2011 to construct two linear and spline models that utilize up to 6 months of historic eGFRs, or logarithm of eGFRs (log‐eGFRs), for prediction of CKD progression to ESKD.^43^ The results of the model were integrated in the CKD Forecaster Tool used at the point of care for nephrologists in clinical decision support system. This helped in‐patient education and care planning for the transition from CKD to ESKD. As reported in a congress abstract, nephrologists who used the CKD Forecaster Tool had less patients transitioning to HD with a central venous catheter.^44^

### 4.2 Treatment aid

#### 4.2.1 Treatment and drug prescription

Prescription of drugs, such as erythropoietin, in patients with ESKD by clinicians is both time‐consuming and error prone. Automation of part of the prescription process could increase efficiency and improve patient care. Several approaches have been published in the literature to reduce erythropoietin dose and increase the percentage of patients within target.^45^ One example is adoption of ANN for anemia management, which was able to increase the percentage of patients in target while reducing hemoglobin variability and erythropoietin dose.^46^

Understanding which drugs are most appropriate for certain patient categories is another area where historic data can guide a decision‐making process for the clinicians. For example, informed by results of virtual clinical trials utilizing advanced physiology‐based mathematical models of parathyroid gland biology, an LDO of FMC, an integrated kidney disease care organization, has afforded nephrologists working in its clinics the opportunity to prescribe off‐label 3× weekly directly observed in‐center administration of cinacalcet as an alternative to daily dosing.^47^, ^48^ Subsequent observations in currently over 11 000 patients indicate that 3× weekly in‐center administration of cinacalcet is noninferior to prescribed daily cinacalcet in controlling parathyroid hormone levels, corroborating the virtual clinical trial results.^49^ Although speculative, efforts like this may potentially further optimize and personalize the treatment of secondary hyperparathyroidism, as well as expand the understanding of the debated influence of mineral bone disorder medications on hard outcomes.^50^, ^51^ In oncology, several studies show successful predictions of which patients would respond to immunotherapy using AI algorithms.^52^, ^53^ Further, ML algorithms have been used to predict which medications would work for which patients with mood disorders.^54^

#### 4.2.2 Identifying medical errors

Although there are not many references in literature on the use of AI in identifying medical errors in a nephrology setting, it is important to highlight how it can be used. Medical errors are a third leading cause of death in the United States; in 2016, they contributed to more than 251K deaths in the United States alone and accrued $17.8 billion dollars in unnecessary spend.^55^, ^56^ Different causes of medical errors exist, such as (a) complexity of the health‐care system; (b) system and process design issues; (c) competency, education, and training; and (d) human factors and ergonomics.

Traditional approach to correct medical errors is to create new rules and procedures that need to be utilized in a health‐care setting.^57^ However, data‐driven, AI approaches can also be applied particularly when historic evidence already exists. Most common application of AI in minimizing medical errors is to guide what therapeutic approaches may or may not be ideal for a given patient. Paredes et al^56^ explored this in the context of US intensive care units and concluded that ML could aid physicians by providing better predictions about the effect of certain treatments and the likely evolution of sepsis patients.

Further, ML algorithms can assist in guiding decisions where complex, time‐dependent, or uncommon medication interactions are at play (such as drug‐drug or drug‐allergy interactions, therapeutic duplication, etc). Traditional rule‐based decision support systems may be insufficient to resolve such issues. AI and technology solutions are likely to be best fitted in these applications.^58^ Specific examples of these applications have been successfully demonstrated by prediction algorithms developed at Stanford University.^59^ Many technology companies have services that support physicians as they interact with their patients' data that may assist in minimizing medical errors.

Outlier management and outlier detection can also assist with minimizing medication errors. This can be completed through AI‐driven algorithms or through Clinical Decision Support models. A team at the Brigham and Women's Hospital evaluated a medication error detection system that uses a probabilistic ML model to identify prescriptions that are outliers based on populations of patients in their EMR system with similar characteristics.^60^

### 4.3 Identifying patterns

#### 4.3.1 Identifying phenotypical patterns

In patients with ESKD, several patterns, such as the malnutrition‐inflammation‐atherosclerosis syndrome, have been discovered by traditional statistical methods. It has thus increased our pathophysiological understanding and were shown to be strong prognostic indicators. Recently, studies have shown that fluid overload also can be part of a pathophysiologic spectrum including malnutrition and inflammation.^61^, ^62^ The concomitant presence of these three risk factors yielded a near six‐fold increase in mortality risk. However, unlike other chronic diseases, pattern detection based on UL techniques has not yet been published in nephrology. In patients with heart failure with preserved ejection fraction, three different phenotypical patterns were identified based on clinical, laboratory, and echocardiographic parameters by agglomerative hierarchical clustering. These clusters differed greatly in mortality risk. In cardiology, the use of UL techniques to detect phenotypical patterns was termed “phenomapping” by the authors.^63^ Another example, infection medicine is based on k‐means clustering on a cohort of patients with sepsis. Four different phenotypes were observed with a distinct difference in outcome, of which one was characterized by older patients with more chronic illness and renal dysfunction (β phenotype). The highest 28‐day mortality (40%) was observed in the δ phenotype, characterized by patients with septic shock and liver dysfunction, as compared to 13% in the β phenotype and 5% in the α phenotype with the lowest risk.^64^ Another study identified different metabolic clusters based on k‐means clustering including a set of clinical parameters and biomarkers in older adults without diabetes. In the clusters characterized by lower eGFR and albuminuria and the cluster with the highest inflammation, the risk of cardiovascular endpoints was comparable to the diabetic cluster.^65^ Whether phenomapping in different diseases has relevance for personalized treatment prescription needs to be addressed in future trials.

#### 4.3.2 Identifying unknown comorbidities

In addition to making predictions about the future, the power of AI can be utilized to comb through vast amounts of information to uncover hidden patterns in high dimensional data otherwise too complex to identify manually. While an incredible resource of clinical data, EMR consist of both structured and unstructured data, with contributions often added by multiple care providers with different documentation styles and levels of thoroughness. Variations and inconsistencies in a patient's record likely increase with the complexity of their health.

One area of concern involves patient comorbidities. ESKD patients with multiple medical comorbidities face decreased survival likelihoods.^66^, ^67^ Prognostic comorbidity indexes indicating patient mortality risk have been used and adapted for renal replacement populations,^68^, ^69^, ^70^ which highlight the critical role that comorbidities play in the complexity of a patient's health picture. In addition to prognostics, comorbidity information is a necessary component involved in medical billing. Medicare's bundled fee‐for‐service coverage for beneficiaries with ESKD includes payment multipliers for patients with complex health pictures based on specific comorbidities. In order to receive appropriate payment for the extra level of support and services tied to these populations, comorbidities must be properly documented in medical records. In nephrology, one LDO within its integrated kidney disease care organization addressed this clinical need by using ML to find patterns in physician notes common across diseases to identify potential undocumented comorbidities or to remove comorbidities that are unlikely to exist.^71^ Using lab test results, NLP of physician notes, and demographic information, the LDO was able to improve coding over the previous method of randomly chosen manual medical record reviews.

#### 4.3.3 Image classification for arteriovenous fistula aneurysm and biomarker fingerprints

The Renal Research Institute developed a CNN to automatically classify arteriovenous fistula aneurysm (AVFA) stages. They collected 15‐20 seconds panning videos from 30 patients to train a CNN model. CNN was able to automatically classify AVFA stages with >90% classification accuracy. As reported in a congress abstract, using this model in a clinical application will reduce workload for physicians, provide timely AVFA diagnosis, and improve patient care.^72^

Advances in biochemical analytics, such as liquid chromatography‐mass spectrometry, provide an unparalleled amount of data from biological samples, giving rise to the rapidly evolving field of metabolomics. A major area of research is to explore if specific compound patterns are correlated with clinical outcomes of interest or if patterns differ between clinical phenotypes. Given the enormous number of metabolites, this question lends itself to the use of AI. Very recently, several groups have successfully applied ML to metabolomics data.^73^ Another example of a potential clinical application for AI in peritoneal dialysis was presented by Zhang et al, who used a combination of supervised ML methods to detect specific immune fingerprints allowing rapid detection of causative organisms in peritonitis, potentially facilitating earlier prescription of specific antibiotic treatment.^74^ This study demonstrated the power of using advanced analytical model for mining complex biomedical dataset where traditional statistical methods fail to yield satisfactory results.

## 5 REFLECTION

Rapid advances in computing, mathematics, and statistics have resulted in the evolution of AI and ML methods. Cloud computing resources might be a more cost‐effective way of analyzing large volumes of data and building ML models. Ideally, ML algorithms should be available for use in the community.

Traditional statistical modeling techniques are most appropriate in building simple predictive models, where one has a well‐defined problem, good observation set, and established knowledge expertise about the strengths and limitations of the outcomes. Furthermore, traditional techniques learn from data which are static in time, and thus tend to “overfit” to their training, and fare reasonably poorly when they encounter anomalous instances.

However, in an ever‐evolving renal care landscape where the problems posed are complex, AI provides several techniques to derive meaningful results. It is very powerful in identifying unknown patterns and anomalies.

Therefore, traditional statistical and advanced AI techniques are both complementary. It is a widely accepted practice to initially build a model using traditional statistical techniques and use that model as a baseline against which AI models are compared against for performance. In a systematic review in the general population, no major differences were found between various advanced AI techniques and traditional statistical modeling techniques in clinical prediction.^75^ A prudent approach is to choose a model appropriate for the problem at hand and not necessarily bias oneself to one methodology. Beyond methodologies, it is important to translate modeling results into actionable decision points for patients and care providers. At present, the use of prediction models in dialysis treatment is still in its infancy and further evidence is needed to identify its relative value.

AI techniques can allow for large datasets to be leveraged with minimal efforts. Such techniques have the power to process large volumes of data to identify patterns and features which may impact the outcome. However, outcome selection and follow‐up timeframes need to be carefully determined to optimize the performance and potential clinical value. AI model that can predict short‐term outcomes may not allow time for interventions to change the course of an event.

AI solutions must follow ethical guidelines and consider at the time of conception whether software programs are medical devices that require formal regulatory pathways and trials.^76^, ^77^ Furthermore, before implementation of AI solutions at the point of care, policies and regulations need to be established for delivery of the outputs to clinicians and patients. Models are never 100% accurate, and thus there will be instances where models will predict incorrectly. In such situations, a precedent of accountability needs to be established. AI solutions should be transparent and traceable. It is important that the predictive models use data collected routinely in standard of care or it will likely produce models that are bias by indication. Teams developing and using AI solutions should be aware of this limitation. Thorough evaluation of the input data variables should be conducted as a key step in the selection of outcomes and the process of building predictive models.

While a lot of emphasis is placed in developing powerful and accurate models, more emphasis should be directed toward building an end‐to‐end team of practitioners in data analytics, data engineering, trainers, care providers, and patients to create effective solutions which would be beneficial for all stakeholders. The effectiveness of the prediction models depends heavily on the ability to use insights to make clinical interventions. On the other hand, interventions need to be thoroughly thought through depending on unique factors driving the clinical outcome and personalized for every patient.

Lastly, AI solutions when implemented at the point of care for nephrologists should be viewed as a clinical decision support tool to extend providers’ insights about the patients. AI is not anticipated to replace providers’ medical decision‐making, but instead assist them in providing optimal personalized care for their patients.

## CONFLICT OF INTERESTS

SC is a student at Maastricht University Medical Center. SC, AL, CM, JWL, SK, FWM, and LAU are employees of Fresenius Medical Care. HZ and PK are employees of Renal Research Institute, a wholly owned subsidiary of Fresenius Medical Care. SC, PK, FWM, and LAU have share options/ownership in Fresenius Medical Care. PK receives honorarium from Up‐To‐Date and is on the Editorial Board of Blood Purification and Kidney and Blood Pressure Research. FWM has a directorship in FMC Management Board, Goldfinch Bio Board, and Vifor Fresenius Medical Care Renal Pharma Board. JPK and FMS have nothing to disclose.

## AUTHOR CONTRIBUTIONS

The interpretation, drafting, and revision of this manuscript were conducted by all authors. The decision to submit this manuscript for publication was jointly made by all authors and the manuscript was confirmed to be accurate and approved by all authors.
